# The Development of a Digital Dysphagia Guide with Care Homes: Co-Production and Evaluation of a Nutrition Support Tool

**DOI:** 10.3390/geriatrics4030048

**Published:** 2019-08-15

**Authors:** Susan Pownall, Elizabeth Barnett, Julie Skilbeck, Angel Jimenez-Aranda, Sally Fowler-Davis

**Affiliations:** 1 Speech and Language Therapy Department,, Sheffield Teaching Hospitals NHS Foundation Trust, Sheffield S10 2JF, UK; 2 Faculty of Health and Wellbeing,, Sheffield Hallam University, Sheffield S10 2TP, UK; 3 NIHR Devices for Dignity MedTech Co-operative, Sheffield Teaching Hospitals NHS Foundation Trust, Sheffield S10 2JF, UK

**Keywords:** dysphagia, technology, workforce development, older adults, care homes

## Abstract

Good nutrition is a recognised outcome in the health and well-being of older care home residents and dysphagia is a known risk factor associated with under nutrition and poor outcomes. The study co-produced a digital Dysphagia Guide with Care Homes using a consensus method with interviews and focus groups to prioritise the need for information and explore acceptability of an educational tool for care home workers. Evaluation of use, acceptability of design, and content of the guide were completed via remote monitoring. The workforce prioritised the need for training as well as the knowledge and skills in relation to planning resident-centred care and advice on textured diets. The technology was a means of offering ‘bite-size’ learning to enhance planning for nutrition across the whole organisation including managers, kitchen staff, and care workers. The Guide to Dysphagia was produced on a tablet and piloted in four care homes over 12 weeks, by 57 staff. Integrated analytics allowed user activity to be monitored. Findings showed that 73% of respondents reported the guide helped them in their job. Additionally, 88% of respondents stated they would recommend the guide to other staff, with 90% reporting it was easy to use. Engagement with staff and managers in four homes resulted in a co-designed, dysphagia guide.

## 1. Introduction 

The World Gastroenterology Global Guidelines 2014 [1] estimate that dysphagia (swallowing difficulties) that results from a range of medical conditions, such as stroke or degenerative conditions, affects more than 51% of institutionalized elderly patients. In the United Kingdom, the number of older residents experiencing swallowing difficulties is growing year by year as national demographics change and age-related changes occur in the swallowing mechanism due to loss of muscle mass and strength [2]. Over 1.47 m care workers are providing support to individuals with dysphagia who are residing in nursing or residential care settings [3]. 

Person-centered care can contribute to quality of life and well-being of nursing home residents, relatives, and staff. However, there is less written about the operationalizing and implementation of best practices and how it may promote well-being and satisfaction [4]. For example, when nutrition and hydration are well managed, i.e., providing a customized diet and consideration of the individual’s nutritional needs, it makes a vital contribution for people recovering from illness and for those at risk of malnutrition. When managed poorly, it poses a significant threat to patient safety [5]. Optimising nutrition and hydration to people with dysphagia is complex and, if impaired swallowing is poorly managed there is a high risk of the individual developing lower respiratory tract infections and pneumonia [6,7]. Although the risk of pneumonia is a significant problem in older people, care home residents are 10 times more likely to develop pneumonia than older people living in their own homes [8] and it is the second most common infection found in nursing home residents [9,10]. Undernutrition is also common in care home residents, which can result in health consequences such as confusion, dehydration, pressure ulcers, constipation, infections, and decreased overall quality of life [11]. Providing the required daily amounts of food and fluid to individuals who present with dysphagia can be particularly challenging. Although it is difficult to calculate the true cost of dysphagia to the care sector, a recent study from Denmark reported the annual total cost for caring for individuals with dysphagia in three municipalities to be DKK 1.425 billion compared to DKK 1.135 billion for those without dysphagia [12].

Optimising support for residents with dysphagia in a care home setting is complex and multifaceted and includes factors around staff resourcing and skill mix, use of dysphagia protocols, supportive interventions at mealtimes, and effective communication across the wider care home workforce. There is general agreement in the literature on the need for improved training for both qualified and unqualified care staff, in the assessment and management of swallowing difficulties [13]. In particular, the ability of care home staff to detect early signs of dysphagia on an on-going basis is seen as crucial for preventing consequent health risks and decline [14]. The Essential Standard of Quality & Safety Outcome 5 Meeting Nutritional Needs [15] requires care homes to identify and take action on swallowing difficulties, including appropriate dietary provision and support. 

Evidence regarding the method and content of training of care home staff in managing dysphagia and nutritional support of residents is inconclusive. Some studies have focused on specific aspects of dietary provision, e.g., texture-modified diets [16] while others have focused on improving specific health outcomes for older residents, e.g., oral health [14,17] or reduction in chest infections [18,19]. There is also a growing body of evidence exploring approaches to delivering education and training such as ‘train the trainers’ [20], and blended e-learning [21]. Integrated approaches to training, including work-based blended e-learning, have demonstrated sustainability of dysphagia management across an organisation [21,22]. However, literature suggests that training in the care home setting is often a challenge and a rapidly changing care workforce results in any positive training effects often being lost within a short period of time [23]. 

This study sought to identify priorities that care home workers perceived as the most useful to improve practice, recognising the limited opportunity for face-to-face learning and, therefore, developing a novel training intervention. Objectives included the analysis of organisational workforce issues including staff time and availability to attend training, as well as acceptability of knowledge learning and practical demonstration of activities such as preparation of nutritious and attractive meals for people with dysphagia. The study adopted a co-design method and a technological platform to support individual’s differing learning styles. The outcome was an evidence-based tool that supported the management of dysphagia in a care home setting. 

## 2. Materials and Methods

### 2.1. Study Design

The study used an experience-based co-design approach and comprised of two phases including qualitative investigations and consensus on learning need, and then product development and initial trial of acceptability. Ethical approval was secured from London - West London & GTAC Research Ethics Committee (REC reference number 17/LO/0213). The aim of the study was to explore the knowledge needs of the workforce and develop a resource to meet this need, in order for them to be able to identify the presence of dysphagia in their residents and to understand how to manage dysphagia holistically via provision of nutritious and appetising meals and understand techniques to assist their residents to eat and drink safely at mealtimes.

### 2.2. Setting

This two-year study was undertaken in four nursing homes in a city in the north of England. The nursing homes were chosen to reflect a range of organisations including corporately-owned and managed and small-to-medium-sized enterprises (SME) with a known number of residents with dysphagia, registered for nursing and/or residential care. Although the number of residents with swallowing difficulties was not recorded specifically, the chosen organisations were regular referrers to the local Speech and Language Therapy service for swallowing assessments and advice about dysphagia management and staff were known to have regular contact with residents with dysphagia.

#### Phase 1: Development of the Tool

Co-design methods have been variously defined, but, in this case, the ambition was to enable a detailed understanding of functionality of the learning needs of care home staff and modelling of a physical system to convert this into product ‘architecture’ [24]. Using an experience-based co-design process [25], the participants can be involved in all stages or simply offer an interview, but recognise their engagement as valuing the lived experience of receiving or delivering care [26]. Typically, products and services are designed without reference to the knowledge needs of the care home workforce. This may be because the co-design process is challenging, requiring all parties to renegotiate their roles and expectations as part of a reconfiguration of the relationships [27]. 

The initial engagement was undertaken with staff and residents and focus groups were used to understand the care implementation model for dysphagia and to understand areas for improvement [28]. In this case, the speech and language therapist researcher led the recruitment based on participant information that asked care home workers to share understanding of the experience of caring for very frail residents with swallowing difficulties. Informed consent was gained from all participants in the study. For recruitment of residents, we worked closely with the care home team and the individual residents to ensure that an individual’s capacity was considered in the context of their daily performance and the complexity of the decision that the person had to make regarding participating in the research. Knowledge generated between the professional (speech and language therapist) and the care home workforce would commonly involve a privileged role for the technical expertise, but, in this case, it was considered most important to understand user characteristics and privilege information in terms of their users’ interaction [29] because the new knowledge was being used to develop a product for use in daily life.

### 2.3. Qualitative Investigation

A qualitative approach was adopted in Phase 1 in order to investigate the subjective experiences of a range of care home staff in their management of dysphagia [30]. To engage and understand the knowledge needs of care home staff, eight focus groups were conducted with 37 members of the nursing, care assistant team, and catering staff across the four participating care homes. Focus groups offered the opportunity for participants to share and debate their experiences and ideas about dysphagia management [31]. This was considered important as part of the co-design process. The group sizes ranged from 3-7 participants (2 groups n = 3, 2 groups n = 4, 2 groups n = 5, 1 group n = 6, and 1 group n = 7) depending on the availability of the participants on the day of each group. To access individual responses and for ease of access for participants, semi-structured interviews were carried out with the care home managers (n = 4) and quality managers (n = 4) from each care home organisation. Semi-structured interviews were completed with six residents. Interview guides and topic guides for the focus groups were developed following a scoping review of the literature and following a discussion session with the speech and language therapist (SLT) who works in the local care homes and who had expertise in the area of dysphagia and understood the workings of the chosen organisations. The moderator for the focus groups was a SLT from the research team (EB). A second member of the research team took notes during the groups and summarised the issues raised at the end of each session (SP/JS). Two members of the research team carried out the interviews (EB/JS). All the focus groups and interviews were audio-recorded and transcribed verbatim. Interview and focus group data was coded and inductively analysed using Framework analysis (Ritchie and Spencer 1994) [32]. Themes were generated by the research team around practice patterns and barriers for implementing good practice into daily routines. The transcripts were read by three members of the research team (SP, EB, JS) and the texts were coded manually, which built an inductive framework. The interview questions and topic guides provided an initial basis for coding and generating themes. As part of the iterative process of analysis, the themes were reviewed by the three research team members and categorised into meta-themes, which were then defined and named.

### 2.4. Nominal Group Methods

The four themes were then prioritised using a nominal group method [33] that involved bringing the care home focus group participants together and developing a deeper understanding of their knowledge needs in practice. The principle of this consensus-based method is that ‘experts’, in this case, care home managers, cooks, and care workers, are able to use a structured and step-wise approach to discuss and refine thematic classifications [34]. The method has been developed to ensure validation by the study participants and where interpersonal, and multi-disciplinary, perceptions may exist and where there may be little analytic understanding of best practice [35]. There were two consensus workshops held representing the views of a range of the workforce (n = 12 and n = 11), involving specific ranking of the most, to the least important factors that influence best practice and person-centred dysphagia management. Fourteen participants had taken part in the previous focus groups. In each case, the care home workforce participants were presented with the findings and then given colour-coded sheets that reflected aspects of the themes derived from the qualitative study. They were asked to familarise themselves with the themes and then to rank them 1 to 10. This includes aspects of the dysphagia management process that were most necessary and important. This data was collated during the event and participants were asked to verify the prioritisation for the purpose of informing the design of the training tool. 

Lastly, the research team was able to construct a knowledge model and share findings with the technology designers to develop and test an interactive user interface. The visualisation process was undertaken on paper with explanation about the access to relative types of information. For example, knowledge about dysphagia, governance, and regulatory information with links to video and recipe materials that cooks and caregivers could access to support residents at meal-times, either by making meals or by feeding and eating support. Principles of the co-design included the need for management of the food provision process, recognising that, for kitchen staff to produce specialist textured meals, which are nutrionally complete and appetising, this requires the purchase of suitable foods, design of specific meal plans, and skilled techniques. This phase of the co-design helped distinuish between operational processes for personalising eating and drinking and also the strategic management required. This includes the policy and governance associated with planning and purchasing food and the preparation of diets.

### 2.5. User Testing

The user interface for the Dysphagia Guide was developed by a design agency. In order to understand how real users experienced the interface, a usability testing methodology was used. Two rounds of moderated testing were done, with participants looking at images of some screens from the application in a tablet and answering a series of semi-structured questions. Participant responses were transcribed and analysed per screen to generate suggestions for improvement. The implementation of usability testing during the initial development phases supported the identification of important considerations for future users of the application to allow them to navigate the information successfully and use the features of the application in a succesful manner.

### 2.6. Technology Development

The dysphagia guide was developed in collaboration with a digital health company. Agile methodology [36] was adopted for the software development, using incremental and iterative work sequences with a focus on rapid delivery. This methodology ensured value was optimised throughout the development process life-cycle, and included a modularity structure, consideration of adjustments needs, and close collaboration with end users. 

Apache Cordova tools [37] were used to develop a hybrid application. This approach enabled the generation of the application for iOS and Android tablets with a single codebase, with a faster and simpler development using HTML5, CSS3, and Javascripts, and a rapid and easier software maintenance.

An app analytics service was integrated in the software to gather accurate data about the activity of the users. Frequency and duration of visits to each page in the Dysphagia Guide was monitored and stored remotely in the cloud to analyse user behaviour and gain insight for future improvements of the solution. 

At the end of the 12-week pilot period, participants were asked to complete a short questionnaire about the user experience and satisfaction using the guide. Questionnaires included 12 questions in the following areas: design, content, usability, accessibility, and impact.

## 3. Results

Four key themes were identifed from the interviews and focus groups: Training, Food Production and Presentation, Quality and Safety, and Workforce.

Training: Access to training and education varied across the care homes. In particular, there were limited opportunities for catering and kitchen staff to develop knowledge and skills regarding food management. Most participants perceived that e-learning packages were limited in supporting the management of residents with dysphagia.

‘I mean I’ll hold my hands up, when I go on that training, some of these are saying this section will take 15 min to read, this one will take 20 min to read, this will take 40 min to listen to or read. You haven’t got time for that. I skip to the assessment and just go on to the general knowledge, what I know, I just hope that I pass at the end’.

(HCA: Focus group 1)

‘There’s an awful lot of online training that I’ve done and a lot of it unpaid, sadly, but there we are. But I’ve watched, you know, endless hours of online training and I’m not saying we want more, by the way, there isn’t a video in the world that will tell you how to help residents eat their breakfast; you have experience, isn’t it’

(HCA: Focus group)

Informal training was most valued by health care workers, such as buddy systems, and ‘flash’ training, as described by a focus group participant:

‘When we are buddying people or introducing them to the job, we do, specifically when we are giving out meals, we are saying to people, you know, you do not just slop food on somebody’s plate; it must look presentable and nice because you are giving it to somebody to eat. I would not eat that! You do not do that in your own home. So, we do train people to be aware of these things’.

(HCA: Focus group 3)

Food Production and Presentation: Lack of choice regarding meals was identified as an issue across most of the care homes, such as the mis-match between central menus and what could be provided locally. There was also variability in the quality, provision, and preparation of texture modified foods, including the use of finger foods.

‘Because, like the lady upstairs, she still does not eat well. And I do not know whether it is because of the look of the food, or she does not like it. She does not really tell you why or whether it is the fact that it is blended. But because the dietitian is so hard to get hold of, you just sort of try different things all the time, whereas they may be able to give you a way of it tasting better or a better texture’.

(Kitchen assistant: Focus group 3)

‘I find the people that are on the modified diet, they have dementia, so to actually ask them they have no idea what you’re talking about. So, I find we have to speak for them when it comes to their meals. But I find it’s repetitive what they have. They have mash, corned beef, and gravy, cheesy mash and beans. But I find it’s a lot of mash. So, it’s quite limited for choice’.

(HCA: Focus group)

Quality and Safety: It was clear that all participants were aware of how to recognise and identify the signs of swallowing difficulty. A ‘first aid’ approach to managing risks was widely used when appropriate, as indicated by the following participants.

‘Some of the more common symptoms are the fact that people are coughing after drinks, or care workers have noticed that they are struggling with food and things and you do start with the initial thing of like looking at their mouth, and whatever, to see if they can eat and then we will pick it up from there’

(Manager: Interview)

‘Staff usually come and tell me, I have just noticed this gentleman’s been coughing and its soup. So, I will look is that the first instance or is there any other staff noticed and they will say well actually, it has been a couple of days, so at that point, it is usually me who does all the speech and language referrals’

(RN: Focus group)

Using a ‘first aid’ approach was important since residents themselves were not always aware that they had difficulty swallowing. One resident who was interviewed stated he had no problems with swallowing but then went on to say, ‘they have given me thicker tea, it has really stopped me spluttering’.

The importance of developing a relationship with the resident was highlighted and this contributed to a person-centred care in the management of dysphagia. Many strategies for ensuring safe mealtimes were shared, including, pacing, resident position, and appropriate cutlery. These all contributed to maintaining safety but also dignity.

‘I make sure they have got enough time between each spoonful. Make sure you have not got too much on your spoon and you are not over forcing them. I think it is positional as well. Rather than we feed them like that, do you know what I mean? Making sure the food is not too dry, they are having a drink in between’.

(HCS: Focus group)

Workforce: The need to develop innovative approaches to enable maximum support at meal times was important. Participants highlighted meal times as ‘pinch-points’ where there was not enough staff across the care home to support residents to eat and drink. In some care homes, there had been a proactive approach to recruitment and retention of staff, such as flexible approaches to contracts and shift patterns.

‘Agency staff, well, we have had agency staff to cover sickness, annual leave, and we have had quite a few agency staff but there is something positive that has come out of that, because our company had started their own agency, so we know what training they have done’.

(Manager: interview)

Evidence of policies to support the care of residents with swallowing difficulties was variable across the care homes. There appeared to be nutritional policies, but it was not always clear whether these included dysphagia management.

‘We have a nutrition policy. I do not know if there is - I would imagine that dysphagia is in it. I do not think we have got a dysphagia policy as such, no. I do not think we have. But without looking at the system, I have to say that I do not think there is anything’.

(Interview: Quality Manager)

At times, the participants perceived that there was a mis-match between central policies and care home practice. Some care homes had introduced dysphagia champions to manage this.

### 3.1. Consensus Building and Co-Design

The priorities were identifed as follows:

1. **Training**—most staff ranked knowledge and skills for safe dysphagia management as a priority including the need for practical mealtime advice and guidance and practical methods for maintaining skills including a ‘champion’ or in house expert, to offer ‘bite size’ prompts.

2. **Food Production and Presentation**—Second, the ranking identified concern about the availability of textured diets and the inclusion of catering staff in dysphagia management including ordering and presenting a variety of options and understanding the negative effect of limited meal opportunities.

3. **Governance and Safety**—Ranking third was the lack of data and consistent management of the dysphagia standards and the need for policy associated with organisational practice, including operational management, budgeting for appriopriate foods, and standardising recording methods for weight loss and amount of meals consumed.

4. **Workforce**—Lastly, the groups ranked workforce as a factor, highlighting mealtimes and refreshment breaks as ‘pinch – points’ in the working day with the increased workload on caregivers requiring specific deployment of staff to provide both a social dining experience and the individualised assistance for a large proportion of residents who are required to eat and drink.

### 3.2. Usability Testing

The first round of usability testing focused on feedback on the intended purpose of the screens and how apparent this was to participants, general feedback on the initial designs, including look and feel, colour scheme, overall design, readability of text, format of articles, and interpretation of pictograms and graphics. Participants were free to give comments about other topics if there was anything else that stood out to them. Three testers were recruited and their feedback was used to update the first version of screen graphics and develop the first prototype.

For the second round of usability testing, six participants were recruited, all of whom had experience of working in care, to look at the updated screen graphics, and give their feedback. Questions were based around the home page, the contents pages for each section, and a food article page. Screens were displayed in a Samsung tablet with an Android operating system. Questioning was semi-structured, but mainly focused on the presentation of the screens, along with some questions based on navigation around the application. Feedback provided was used to update the screens and develop the final product.

Content was reviewed by three healthcare professionals via a web link. Detailed feedback was provided regarding the text, grammar, colours used, website links embedded, images added, typographical errors spotted, whether the video worked, structure of the sections, text to rephrase, and ease of navigation through the pages. 

### 3.3. Final Version

**Figure 1:**
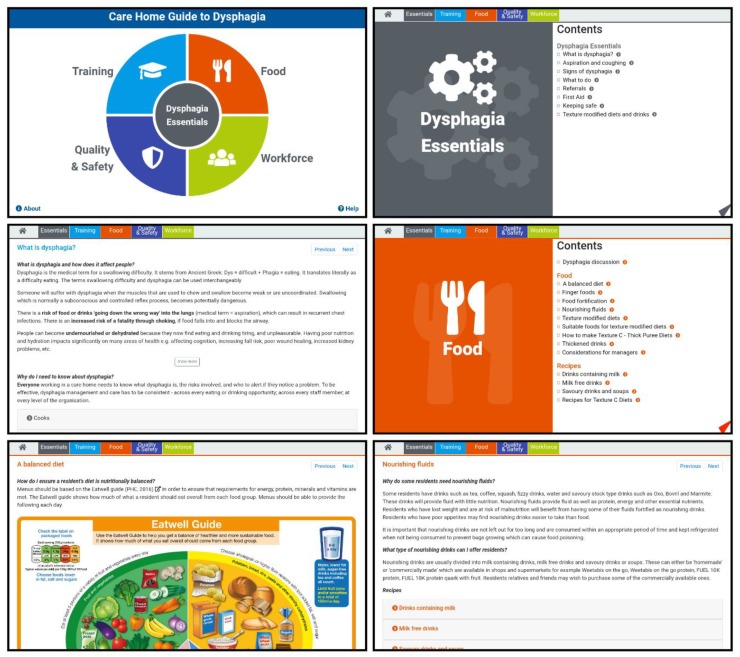
Screenshots of the dysphagia guide.

The final product with the reviewed content and designs was presented as an app compatible with Android and iOS tablet devices. The initial first page in the app presented a coloured design with direct links to the four themes, plus a link to the Essentials section in the centre, and two small links to ‘About’ and ‘Help’ sections. All the other pages included a combination of text, images, and videos with a top banner to facilitate the navigation in the app. Sample screenshots are presented in Figure 1.

### 3.4. Technology Evaluation 

The aim of phase 2 was to test the usability of the hardware and to validate the content of the dysphagia guide in care home practice.

The care home guide to dysphagia was piloted in the four participating care homes by 57 staff. The same participants who had been involved in the tool development phase piloted the tool where possible and held a range of roles within the homes. None of the six residents were involved in the piloting. Each home was given two tablets to use over a 12-week period. Since the digital guide included dysphagia knowledge, videos and information about dysphagia management and was also a novel digital device that was accessed via a tablet, there were several elements of acceptability to assess. For this initial stage, it was important to understand which knowledge elements were more interesting and whether the design was acceptable; allowing the care home workforce to access the knowledge that they needed in practice.

### 3.5. Findings of Digital Analytics and Questionnaire

During the pilot, the pages were displayed a total of 1913 times. More than half of these displayed were in two sections: Food (33%) and Essentials (22%). All the pages were visited at least twice during the pilot, and 77% of the pages were visited more than 10 times. Besides the five content pages (1 for each section + main content page), the most visited pages are shown in Table 1.

On average, each user spent 19 minutes and 25 seconds per session visiting 28 screens, resulting in 42 seconds spent per page. This is a significant amount of time, which demonstrates their interest regarding the content included in the dysphagia guide. The two pages where users spent more time were ‘Recipes from Care Homes’ with an average of 3 minutes and 18 seconds per visit and ‘Dysphagia discussion’ with an average of 2 minutes and 24 seconds per visit. It is notable that almost 50% of the total time users spent with the Guide was in the ‘Food’ section.

There was no direct relationship between time spent on a page and the inclusion of a video in the page. From the top 10 pages where users spent more time, four of them did not include a video (‘Recipes from Care Homes’, ‘Keeping Safe’, ‘Signs of Dysphagia’ and ‘Pinch Points’). 

The dysphagia guide includes 52 pages of which 23 pages (44%) received feedback from at least one user. Users provided feedback 111 times (6% of pages displayed). Additionally, 52% of the 110 positive clicks were on pages included in the ‘Essential’ section, 31% in ‘Food’, 14% in ‘Training’ and 3% in ‘Workforce’.

Of the 57 participants who were given a questionnaire, 36 (63%) were returned. 

86% of the sample was female and 14% was male. The age of participants ranged from 16 to over 55 years with 25% of the workforce being in the older age group. Respondents held a range of roles within the homes including: Management/ training (26%), Nurse (8%), Health Care Assistant (47%), Cook/Kitchen staff (17%), and Other (2%). Years of experience varied from 6% of respondents having less than one year of experience and up to 42% having more than 10 years of experience working in care settings.

Results from the questionnaire are shown in Table 2. 73% of respondents reported the guide had helped them in their job and, in particular, 80% felt the videos were helpful. 90% reported the guide was easy to use and 93% felt the organisation of information was clear. Out of all respondents, 88% would recommend the guide to other care home staff. However, 25% reported the tablet was not available when they wanted to use it as a result of it being locked in the manager’s office.

## 4. Discussion

The study demonstrated how the co-design process could be used for the development of a digital guide to support the management of dysphagia in care home settings. The methods engaged a multi-disciplinary research team and a group of participants who contributed by describing the reality of managing nutrition and meal times for residents who have a swallowing disorder. The analysis undertaken with the same participants enabled the co-design of a product interface that meant the information was accessible to care home workers and that they could use knowledge about dysphagia, and textured diets as they needed it. The analysis also led to an understanding about the organisational arrangements and how critical these were to improving frontline practice. These experience based co-design processes [38] are an important method to ensure that innovation and particularly product development makes sense to front line staff and that the wisdom from everyday practice is captured for the design. Action methods can be seen to achieve more sustained implementation in care home developments [39,40].

The development of the portable digital guide to dysphagia provides care homes with the opportunity to introduce a more applied, interactive, work-based approach to education and training of the entire care home workforce. The incorporation of videos, text, and photographs was important to cater for a range of different learning styles. Notwithstanding the issues surrounding engagement with e-learning, there is increasing evidence that more traditional blended learning approaches must be combined with work-based learning and actual practical experience [41] and, where it is used, it is more likely to be associated with changes to care delivery and, in some instances, outcomes for patients [42]. During the co-production exercise, it became clear that participants required a resource that would be accessible in the workplace, providing bite-sized learning (BSL) opportunities, as well as catering for a range of learning styles. It is increasingly recognised that BSL is able to meet the requirements of work-based learners [43,44]. For the participants in this study, the ability to use a device flexibly, in context, in real time, and, on multiple occasions, has the potential to enhance knowledge and skill development in relation to the management of dysphagia in older residents living in care homes. In order to maximise usage more widely and to bring real benefits to the care of residents around eating and drinking, organisational and cultural aspects around acceptance of the digital technology would need further exploration.

The initial testing of the product design, bears out the findings of the consensus workshops and demonstrates that the prioritisation and organisation of the information was a novel approach to technology development, which ensures that the product development and the associated access to information was co-designed to meet resident needs and that which care home staff were required to modify their care activities [45]. The request for information to underpin the skills and practice was an important consideration for the care home workforce who is generally considered to not require technical health knowledge and who is rarely consulted in spite of their intimate knowledge of hands-on care delivered to seriously ill, functionally impaired individuals in their homes [46]. A co-design approach enabled the research team and product design to identify competencies, training modalities, and frequencies of use, and to identify the knowledge and information the care workers would find most beneficial to their roles of supporting their residents. The content of the pages most visited in the guide support key issues highlighted in other studies where it is recognised that training in preparation of modified texture diets and fluids for residents with dysphagia should be available and, where it is given, it is seen as valuable with demonstration evaluating very positively [16]. Inclusion of short videos in the guide for preparation of modified diets and fluids reflected this finding and was the topic visited most frequently by staff, which supports the notion that training should be provided to the staff responsible for designing menus and preparing the food, in practical skills and in methods driving the addition of nutrition to pureed diets and making meals more attractive [47,48]. Currently, the guide does not conclude with a ‘test’ of knowledge acquired during its use. However, this would be something to consider in any subsequent versions.

As expected, our findings show that e-learning is used within the care homes to provide education and training for staff. This is not unsurprising given that e-learning reduces the costs of training from an organisational perspective as well as enhancing the monitoring of staff accessing and completing training [49,50]. However, our study highlighted that participants were ambivalent toward the use of classroom and distance-based e-learning, which challenged how traditional approaches to ‘blended’ e-learning are used in the care home setting. A recent study by Keenan et al. (2018) [51] exploring the implementation of e-learning and e-tools for care home staff supporting residents with dementia demonstrated that the use of these approaches was limited. In our study, organisational, personal, and professional barriers were identified as reasons for this ambivalence, such as lack of digital literacy, limited time to access online materials, and managerial support. Some participants had no access to education and training, which is specifically related to kitchen and catering staff. Alongside this, our findings highlighted that e-learning packages did not generally focus on the assessment and management of dysphagia, and that training in this clinical area was not mandatory for staff. Relevant education and training is imperative to ensure that care homes are identifying residents with dysphagia and managing nutritional support. There are national guidelines and resources to support this (ESCSO Outcome 5) [15]. 

### Methodological Considerations

An important component of using an experience-based co-design process is recognising the value of engaging with the lived experience of those receiving or delivering care [26]. In this study, we sought to capture the experience of a range of participants. A strength of our study was the involvement of care home staff across all stages of the study design. Not only did this contribute to the development of an appropriate evidence-based learning tool that supported the management of dysphagia in the care home setting but it made staff feel valued in that their knowledge and experience made a valid contribution to the design and implementation of the tool. However, a limitation of our study was the inclusion of only a small number of older residents. Therefore, it could be argued that the voice of the older person is missing within the process. Likewise, including the voice of families of the residents would also have added value. We were reliant on the support of care home staff to access the older residents and their families and, therefore, it is likely that recruitment to the study was influenced by ‘gatekeeping’ such as where personal judgement about who should be involved was made (McKeown et al. 2010) [52]. However, it was important for the research team to respect the views of the care home staff, in particular that some form of protection was necessary.

The digital format of the dysphagia guide facilitates scalability and wider deployment of the technology and ensures a unified approach for all users [53]. However, putting technology into the care home will not automatically translate into benefit, without the people involved being supported through an integrated change program. The program should include creation of a compelling vision for the future alongside technology integration with current work processes. 

## 5. Conclusions

A digital dysphagia guide was co-designed with care home staff to provide a knowledge resource for organisations, managers, and care workers, increase knowledge, confidence, and skills in supporting their residents with dysphagia, and to use best practices aligned with the international standards of evidence for dysphagia management. The co-design processes took place over a two-year period. The processes also engaged a number of key staff roles across the care organisations and enabled the development of the product and initial testing of the tool to confirm the utility in the care home setting. Plans are currently in place to refine the guide in response to feedback from the users in this study, such as the addition of more videos and updating of the diet descriptor terminology to those of the International Dysphagia Diet Standardisation Initiative [54]. A further study is planned to scale-up the implementation of the dysphagia guide and evaluate its impact in collaboration with a greater number of care homes and their workforce. This next stage of the process will also include refinement of the technology to optimise accessibility of the guide by increasing the range of platforms, i.e., mobile phones and personal computers from which the guide can be accessed by users.

## Figures and Tables

**Table 1:** Most visited paged in the digital dysphagia guide.

Page	Number of Visits
‘What is dysphagia’	60
‘Texture modified diets and drinks’	42
‘Thickened drinks’	37
‘Suitable foods for texture modified diets’	36
‘Online resources’	32
‘How to make Texture C’	30
‘A balanced diet’	29
‘Aspiration and coughing’	28
‘Finger Foods’	26

**Table 2:** Rating derived from 5-point Likert scale: 1: strongly disagree, 2: disagree, 3: neutral. 4: agree, and 5: strongly agree.

Question	Mean Rating and Standard Deviation	Percentage of Responses with Rating ≥ 4
The guide has helped me in my job with regards to residents who have swallowing problems	3.77 0.86	73%
The information was presented in a way that is easy to understand	4.10 0.55	90%
It was easy to find the information I needed	3.90 0.67	72%
The organisation of information on the tablet is clear	4.10 0.48	93%
The video clips helped explain issues and demonstrate practical skills	4.03 0.67	80%
The tablet is a convenient way of accessing information about dysphagia	4.07 0.58	87%
The tablet was always available when I wanted to use it	3.33 1.24	52%
The tablet is easy to use and navigate	3.97 0.61	80%
Overall, I am satisfied with the dysphagia guide	4.03 0.86	78%
I would be likely to recommend this guide to other staff working in care homes	4.16 0.63	88%
